# Adverse Childhood Experience, Parental Bonding, and Fatherhood as Parenting Vulnerabilities to Social Anxiety Severity

**DOI:** 10.3390/ejihpe14070137

**Published:** 2024-07-18

**Authors:** Rasoul Heshmati, Nazanin Seyed Yaghoubi Pour, Parisa Haji Abbasoghli, Mojtaba Habibi Asgarabad

**Affiliations:** 1 Department of Psychology, Faculty of Education and Psychology, University of Tabriz, Tabriz 5166616471, Iran; nazaninyaghoubi1400@ms.tabrizu.ac.ir (N.S.Y.P.); abbasiparii@gmail.com (P.H.A.); 2 Department of Psychology, Norwegian University of Science and Technology, 7491 Trondheim, Norway

**Keywords:** adverse childhood experience, social anxiety, parenting vulnerabilities, parental bonding, fatherhood, anxious thought, trauma

## Abstract

**Background:** The present study aims to elucidate the association between adverse childhood experiences, parental bonding, fatherhood, and social anxiety symptoms among emerging adults within an Iranian context. **Methods:** This prospective cross-sectional study utilized self-reported assessments to evaluate fatherhood, parental bonding, anxious thoughts, and childhood trauma. The study was administered to 242 university students exhibiting social anxiety symptoms. Among the participants, 181 (74.8%) were boys and 61 (25.2%) were girls between the ages of 18 and 29. In terms of educational background, 64.9% of them held a bachelor’s degree, and 35.1% held a master’s degree. A majority of them (84.3%) were of middle-class socio-economic status, 6.6% were of low income, and 9.1% were of high income. **Results:** Analysis via multiple linear regression revealed that individuals with adverse childhood experiences exhibited heightened levels of social anxiety symptoms (R^2^ = 0.32) compared to their counterparts without such experiences. Furthermore, fatherhood (R^2^ = 0.28), paternal bonding (R^2^ = 0.26), and maternal bonding (R^2^ = 0.26) were all significantly and equally associated with variance in social anxiety symptoms. The findings underscored the substantial correlation between ACEs, fatherhood, and both maternal and paternal bonding with social anxiety symptoms in adulthood. **Conclusions:** Accordingly, the study emphasizes the importance of thoroughly assessing the multifaceted contributors to social anxiety. Such insights are pivotal for the design and implementation of community-based preventive interventions aimed at reducing the societal burden of social anxiety disorders.

## 1. Introduction

Social anxiety disorder (SAD) is relatively widespread among young individuals, with a lifetime prevalence rate between 3.5% and 9.1% for those aged 10 to 24 in Western nations such as the United States, Germany, and Austria [1,2,3,4]. Epidemiologic research indicates that 36% of adults, particularly youth, meet the threshold criteria for social anxiety disorder [5]. The prevalence of social anxiety in Iran currently ranges from 10% to 40% [6,7]. Based on robust evidence, having levels of social anxiety predispose emerging adults to various negative outcomes such as poor academic performance, low levels of social interaction, susceptibility to depression, suicidality, poor social skills [8,9], high rates of sickness absence, and unemployment [10]. It is also worth noting that social anxiety ranges from an absence of social fear to normal anxiety and shyness, culminating in social anxiety disorder [11]. Thus, given its wide spectrum, substantial negative impact, and high lifetime prevalence, social anxiety disorder as a public psychological concern has received considerable research and clinical attention.

A great surge of research identified adverse childhood experiences (ACEs) as one of the factors leading to social anxiety disorder [12]. ACEs are traumatic events that occur to children and adolescents under the age of 18 [13]. These events include a wide spectrum of traumas, such as physical and emotional neglect, various types of abuse (physical, sexual, and emotional), witnessing domestic violence, mental health issues, family incarceration, parental separation, and substance abuse [14,15]. Likelihood of encountering ACEs can be influenced by numerous factors, including individual, family, and community circumstances, for instance, living in unstable housing and having parents who have themselves experienced ACEs [16]. Epidemiological studies show that millions of children around the world experience ACEs annually [17]. In Iran, researchers from various fields have increasingly recognized the high prevalence of different types of ACEs [18,19,20]. Research into developmental psychopathology reveals that 28.9% of individuals with psychiatric problems report experiencing ACEs at some point of their lives [14,21,22,23]. ACEs significantly impact an individual’s well-being and development, leading to a range of psychological and psychopathological outcomes [24]. These outcomes include, but are not limited to, depression [25], anxiety [12], post-traumatic stress disorder (PTSD) [26], antisocial personality [27], substance abuse [28], and body image disorder [29]. A meta-analysis study indicated that exposure to any form of childhood maltreatment may more than double the risk of anxiety symptoms in adulthood [30]. Notably, those who encountered three or more of the traumas mentioned displayed higher susceptibility [31,32]. It is also important to note that social anxiety disorder is often comorbid with other psychological disorders, such as body image disorders. For example, research has shown a strong correlation between social anxiety and body image concerns, which can further exacerbate the symptoms of both conditions [29]. Several theories have been put forward to explain the links between ACEs and social anxiety.

The stress sensitization theory suggests that exposure to nearby stressors may increase the likelihood of developing mental disorders in individuals who experienced negative childhood experiences. In other words, that early adverse experiences can sensitize a person to stress, making them more likely to react negatively to stressors encountered in adulthood [33]. Additionally, cognitive theories highlight maladaptive emotion regulation as central to depression and anxiety disorders [34,35]. Emotion regulation involves the methods individuals use to control which emotions they feel, the timing of these emotions, and how they experience and express them. This includes both conscious and unconscious techniques for altering or managing the physical, behavioral, or subjective components of an emotional reaction [36]. On the contrary, emotion dysregulation is marked by challenges in controlling emotional reactions in a manner that aligns with societal norms. It encompasses issues with the strength, duration, and suitability of emotional responses, potentially resulting in ineffective behaviors and psychological discomfort or distress [37]. ACEs can impair the development of secure attachments and healthy interpersonal relationships with parents, which are critical for learning effective emotion regulation strategies. Without stable and supportive relationships, individuals may lack the necessary guidance and modeling for healthy emotional development [38]. Chronic stress and trauma from ACEs can lead to heightened emotional reactivity and difficulties in calming down after emotional arousal. Childhood is the formative period for developing emotion regulation abilities, and any adversities during this period lead to failures in effectively regulating emotions [39]. Emotion regulation also mediates the association between ACEs and social anxiety [34]. Thus, children who experience trauma are prone to subsequent social anxiety symptoms [40,41]. The cognitive-behavioral model of social anxiety disorder also says that the disorder starts when a person is biologically predisposed to it and has negative social learning experiences [42]. In this context, maladaptive thought patterns are distorted perceptions of social interactions, such as anticipating negative judgments from others or exaggerating the potential consequences of social mistakes. These distorted thoughts can result in avoidance behaviors, where the individual shuns social situations to evade the expected negative outcomes. Over time, this avoidance strengthens the fear of social interactions and sustains the cycle of anxiety [43]. According to Bowlby’s theory [44], children develop coping mechanisms to manage stress based on their parents’ and family environment. However, ACEs during childhood can hinder the development of a healthy self-concept, including a sense of self-worth, self-awareness, and self-acceptance [45]. These elements are crucial for individuals to effectively navigate life’s challenges, sustain fulfilling relationships, and pursue personal growth and fulfillment. Consequently, such disruptions may lead to social withdrawal [46].

A substantial increase in international research has supported the correlation between ACEs and social anxiety symptoms across various cultural contexts [47,48,49,50]. To illustrate, a study conducted in Korea specifically investigated childhood financial hardships as a significant type of early adversity. The findings revealed that children from lower socioeconomic backgrounds, dealing with financial difficulties, exhibited heightened vulnerability to mental health issues like social anxiety later in adulthood. Moreover, in Korean culture, there is a prevalent belief that traumatic experiences are shameful events that individuals are often expected to bear in isolation. This cultural belief can further compound social anxiety, as individuals may feel compelled to conceal their struggles and suffer silently. Consequently, the societal stigma surrounding trauma may exacerbate the challenges faced by those already dealing with social anxiety symptoms [51].

Overall, childhood trauma as a strong predictor of social anxiety disorder should not be overlooked; however, positive childhood experiences, such as strong parental bonding, have been identified as a protector against vulnerabilities to social anxiety [52]. Parental bonding refers to an emotional attachment, commitment, and social relationship with parents/caregivers [53]. Positive parental bonding and secure attachment styles with parents play a crucial role in promoting children’s psychological well-being and facilitating their optimal development [54]. When children experience warmth, affection, and consistent caregiving from their parents, they develop a sense of security and trust in their relationships. This secure attachment serves as a foundation for healthy emotional regulation, resilience, and self-esteem throughout their lives [55]. Moreover, children who have secure attachments are more likely to explore their environment confidently; develop positive social relationships; and achieve their full potential academically, socially, and emotionally [56]. Robust evidence showed that poor parental bonding, such as changes in parents’ roles, parenting styles, and overprotection, moderated the effect of ACEs on the child’s anxiety [57,58]. In a nutshell, the parent–child relationship can intensify the traumatic experience, which can result in severe symptoms across the anxiety spectrum [59,60]. The importance of parental bonding in the development of social anxiety disorder and its role as a bridge between ACEs and social anxiety symptoms make it even more important to study it. A child’s ability to form this essential bond largely relies on the quality of care and protection offered by a parent [61]. Abnormal parental bonding, characterized by inadequate care, excessive protection, and heightened criticism, can increase susceptibility to social anxiety or other psychiatric disorders [44,52,62]. Kidd et al. [63], in a systematic review of 57 studies, reaffirmed that individuals with anxiety symptoms reported poor quality parental bonding with lower parental care and higher overprotection than those without psychiatric symptoms. Additionally, a meta-analysis of 53 studies in China (*n* = 26,024) revealed that positive parenting is associated with a decline in social anxiety, while negative parenting is associated with increased social anxiety [64]. Based on Bowlby’s attachment theory [44], the years between infancy and emerging adulthood are crucial for psychological development. Thus, inadequate parental input during this period not only hinders psychological development but also renders the child susceptible to psychiatric disorders [44]. A child with secure emotional bonds also finds herself or himself worthy of affection, as well as having positive interactions with others. Conversely, a child with an insecure bond may struggle with self-regulation, exhibit low self-esteem, or perceive themselves as unworthy, subsequently leading to social anxiety symptoms [65]. Previous literature has converged on the idea that there is a strong association between traumatic events and insufficient parental care [59]. For example, the frequency of ACEs is significantly higher in people who are separated from their parents, have violent relatives, or have any plausible deficits in parental bonding [66]. Therefore, parental bonding is an essential psychological process for a child’s optimal development, particularly in the context of fatherhood [67].

According to the literature, most research has concentrated on the roles of mothers, with few considering fatherhood [68]. Based on the view of Dick [69], fatherhood is an individual’s view of the father’s emotional response and the roles fathers engage in based on the child’s experiences growing up. The responsible engagement of the father in behaviors like participation in academic and sports activities makes children have less threatening perceptions and worries about the surrounding environment by observing their parents’ attitudes and behaviors [70]. This may increase affective cognitions and coping strategies and decrease avoidance strategies in society, which lowers the level of social anxiety [71]. Accessibility to the father is associated with lower levels of social anxiety [72]. Conversely, evidence shows that memories of the lack of a father’s responsiveness in early childhood are associated with depression [73] and social anxiety [74]. A recent study in over 3000 families in the UK found that mental health problems in fathers or the father’s absence from the child may affect the child’s cognition, emotions, and social ability, in addition to affecting the father [75]. Based on social learning theory [76], fathers may serve as models for their children, demonstrating active, independent, and competitive behaviors. Children observe and imitate their fathers, which can influence the development of these traits in the child. Consequently, fathers play a challenging role in fostering a child’s active, competitive, independent, and inquisitive nature, and in helping these traits transition into the social world, especially when young adults move away from home [77]. This differs from the mother–child attachment relationship, which aims to console and soothe offspring [78]. Existing theories propose that the effective dynamic relationship between child and father, referred to as the father–child activation relationship, has the function of pushing the child’s limits and, in this way, can buffer against early separation, strangers, and the novelty of the development of anxiety [79]. Specifically, by pushing a child’s limits, fathers encourage their children to explore and interact with their environment, fostering independence and resilience. This process helps children develop the skills and confidence needed to navigate unfamiliar situations, such as separation from their parents or encounters with strangers [74]. Drawing from attachment theory [44], the father’s unfavorable behaviors (inconsistency in parenting, emotional unavailability, dismissiveness of the child’s emotions, etc.) in the child–parent relationship can create an insecure attachment style in the child, characterized by feelings of anxiety and uncertainty about the availability of parental support. This insecurity can lead to difficulties in regulating emotions effectively. Moreover, according to cognitive-behavioral theory, children learn from their experiences and develop cognitive schemas, or beliefs, about themselves and the world around them. If a child consistently experiences rejection or emotional neglect from their father, they may develop negative beliefs about themselves (e.g., “I am unlovable” or “I am not worthy of support”), which can impact their overall cognitive functioning and emotional well-being [80]. This, in turn, poses a significant obstacle to the emotion regulation process, which plays a crucial role in the emergence of social anxiety [81]. Moreover, based on the emotional security theory (EST), fathers are essential sources of emotional security [82]. Therefore, when their fathers reject them, children suffer from a lack of affection and security, which can lead to feelings of inferiority, anger, and a lack of self-confidence [83]. In contrast, children of accepting fathers showed a strong ability for innovation and emotional security, which is a supporting factor in confronting social anxiety [79].

### The Study Context

In traditional Eastern societies, such as Iran, an authoritarian parenting style (a strict style of parenting that places high expectations on children) is prevalent, resulting in cultural influences that lead fathers to adopt a less engaging and more authoritarian role in the father–child relationship [84]. Time limitations due to employment obligations may restrict fathers’ ability to form relationships with their children, potentially leading to mothers assuming primary responsibility for the direct upbringing of children [85]. In addition, it is the responsibility of mothers to facilitate relationships between fathers and children, which contributes to a variety of positive outcomes, including enhanced well-being and self-efficacy [86]. Accordingly, cultural structures reinforce gender roles and restrict the involvement of fathers in the Iranian context [87]. Evidence showed that a majority of Iranian adolescents reported that their mothers were more familiar with them, provided more guidance, and were more involved in their lives than their fathers. This highlights the critical role of mothers in Iranian childrearing, while fathers’ duties appeared to be weaker [84]. 

The present study investigated the etiology of social anxiety, focusing on factors related to primary family relationships that may hinder an individual’s ability to function confidently in society as an adult. A thorough examination of these factors could aid in disease prevention and prognosis. This study aimed to evaluate the associations between adverse childhood experiences (ACEs), social anxiety symptoms, parental bonding, and fatherhood. To the best of our knowledge, the existing literature lacks research on the role of fatherhood in social anxiety, and this study seeks to provide a new perspective on this issue. Additionally, no research has been conducted within the context of Persian culture examining the simultaneous effects of ACEs, fatherhood, and parental bonds. We formulated several specific hypotheses in light of the broader research on social anxiety. (i) We hypothesized a significant negative correlation between social anxiety symptoms and fatherhood. (ii) A significant negative association between parental bonding and social anxiety symptoms was expected. (iii) We anticipated a significant positive correlation between adverse childhood experiences and social anxiety symptoms.

## 2. Materials and Methods

### 2.1. Participants

A total of 980 students were recruited from various faculties at the University of Tabriz. A total of 270 students fulfilled the subclinical levels of social anxiety, and among them, 28 (10.3%) respondents filled out the questionnaires incompletely. Thus, the final sample included 242 participants. The inclusion criteria were as follows: (a) attending university; (b) having subclinical levels of social anxiety symptoms based on self-reports; and (c) being at the age of 18–29 (emerging adults). The exclusion criteria were as follows: (a) currently undergoing psychotropic psychotherapy or medications at present (to avoid confounding variables that could mask the true impact of study variables). A total of 181 (74.8%) of respondents were boys and 61 (25.2%) were girls between the ages of 18 and 29 (M = 23.74, SD = 2.06). A majority of them (84.3%) were of middle-class socioeconomic status, 6.6% were of low income, and 9.1% were of high income. In terms of educational background, 64.9% (*n* = 157) of them held a bachelor’s degree, and 35.1% (*n* = 85) held a master’s degree.

### 2.2. Measures

***The Anxious Thought Inventory (AnTI).*** One of the most commonly used measures to assess anxiety thoughts is AnTI [88]. In the current study, we used the social worry component of AnTI to measure social anxiety. The AnTI consists of 21 items with several empirically derived components that separately measure social worry (eight items), health worry (six items), and meta-worry (seven items). Social worry measures worry related to social interactions and performance in social settings. Health worry focuses on concerns about personal health and physical well-being. Meta-worry reflects concerns about the uncontrollability and consequences of one’s own worrying. A sample item reads, “*I worry about not being able to cope in life as adequately as others seem to*”, and responses were scored on a four-point Likert scale (1 = *almost never* to 4 = *almost always*). Moloodi et al. [89] have confirmed the psychometric properties of the AnTI in an Iranian sample. Moreover, Cronbach’s alphas for social worry, physical health, and meta-worry were 0.85, 0.81, and 0.75, respectively.

***Childhood Trauma Questionnaire (CTQ).*** The CTQ was developed by Bernstein [90] and consists of 28 items (including 25 clinical and 3 validity items) that assess the severity of different types of childhood trauma. The CTQ has five subscales, namely, emotional abuse (5 items), physical abuse (5 items), sexual abuse (5 items), emotional neglect (5 items), and physical neglect (5 items). Participants respond to each item in the context of “*when you were growing up*” and answer according to a five-point Likert-type scale ranging from 0 = *never true* to 5 = *very often true*. For example, one of the items reads, “*People in my family said hurtful or insulting things to me*”. Higher scores indicate greater childhood trauma, also confirming the psychometric properties of the Iranian version of the CTQ. Cronbach’s alphas of the total scale for physical, emotional, and sexual abuse, as well as physical and emotional neglect, were 0.89, 0.80, 0.86, 0.91, 0.84, and 0.78, respectively.

***Parental Bonding Instrument (PBI).*** The PBI [91] measures perceived parental behavior during the first 16 years of life [92]. It consists of two separate forms for the mother and the father. The present study solely utilized the mother form, evaluating the parental relationship solely from the perspective of the mother. The PBI has been compiled by Parker based on Bowlby’s attachment theory and asks respondents to recall how their mothers treated them in the first 16 years of their life by answering multiple choices rated on a four-point Likert scale from 0 = *almost like that* to 3 = *almost not like that at all*. PBI has 24 items and 4 factors, namely, care, overprotection, indifference, and encouragement of autonomy. As an example, one of the items states, “*My mother spoke to me in a warm and friendly voice*”. Behzadi and Parker [93] evaluated the validity and reliability of the Iranian version of the PBI. In their research, the Cronbach’s alphas for care, overprotection, indifference, and encouragement of autonomy in the mother’s form were 0.83, 0.88, 0.79, and 0.80, respectively.

***Fatherhood Scale (FS).*** The FS scale was developed by Dick and consists of 64 items in the form of descriptive statements to measure the type of relationship adults had with their fathers while growing up. The participants should answer based on their perceptions about the quality of the emotional relationship with their fathers during the developmental period. For instance, a sample item reads, “*My father comforted me when I was feeling bad*”. The responses are rated on a five-point Likert scale from 0 = *never* to 4 = *always*. The FS assesses the father’s engagement on nine subscales, including positive engagement (5 items), positive emotional responsiveness (13 items), negative engagement (11 items), moral father role (5 items), good provider role (4 items), and gender role model (6 items). Amlashi, Hosseinkhanzadeh [94] evaluated the psychometric properties of FS in an Iranian sample. Cronbach’s alphas for the total scale, positive emotional responsiveness, moral father role, father’s positive engagement, good provider role, negative engagement, gender role, androgynous role, responsible father, and accessible father were 0.96, 0.96, 0.85, 0.67, 0.79, 0.78, 0.82, 0.89, and 0.84, respectively (see Table 1 for the descriptions and Cronbach’s alpha of the tools for the original and current studies).

### 2.3. Procedure

A university-based ethics committee approved the data collection and study. Ethical considerations were clarified (such as voluntary participation, privacy, anonymity, and confidentiality). Before participating, each respondent received information about the study and signed an informed consent form. They were informed that participation was voluntary and that they could withdraw at any time. The questionnaires were anonymous, and no participant responses were discussed individually in this study. Before completing the AnTI, CTQ, PBI, and FS, participants were asked to respond to demographic questions regarding their age, gender, level of education, and socioeconomic status; no compensation was offered.

### 2.4. Statistical Strategy

Descriptive statistics for the variables were calculated using SPSS version 24. Mul-tiple regression analysis was conducted, and the assumptions of multiple regression were verified. Less than 3% of the data was missing, and regression estimation was used to impute the missing values. Outliers were identified using the squared Mahalanobis distance (D2), resulting in the exclusion of 28 individuals with extreme D2 values, leaving a final sample size of 242 participants. Normality was assessed using the coefficients of skewness (Sk) and kurtosis (Ku), and all variables demonstrated acceptable normality. The dependent and independent variables were linearly correlated, residual terms were uncorrelated, no heteroscedasticity was observed, the error distribution was normal, and there was no evidence of multicollinearity. The assumptions for regression analysis were met. A *p*-value of ≤0.05 was used to determine statistical significance. To investigate the associations between adverse childhood experiences, parental bonding, fatherhood, and social anxiety levels, a series of multiple regression analyses were performed.

## 3. Results

As shown in Table 2, generally, maternal bonding, paternal bonding, fatherhood, and adverse childhood experiences can explain 39% of changes in social anxiety (F = 37.41, *p* < 0.05). The results related to regression coefficients indicate that maternal bonding (t = −3.13, β = −0.20 *p* < 0.05) and fatherhood (t = −2.35, β = −0.12, *p* < 0.05) negatively predicted social anxiety. That means by increasing one unit in variables like maternal and paternal bonding and fatherhood, the amount of social anxiety decreased by 0.20, 0.29, and 0.30, respectively. Moreover, adverse childhood experiences (t = 3.48, β = 0.22, *p* < 0.05) positively predicted social anxiety. In other words, by an increase of one unit in adverse childhood experiences, the number of social anxieties increased by 0.22 units. 

Based on Table 3, components of maternal bonding were generally able to explain 27% of changes in social anxiety (F = 21.52, *p* < 0.05). The results of the regression coefficients showed that components of care (t = −5.01, β = −0.24, *p* < 0.05), overprotecting (t = −2.77, β = −0.18 *p* < 0.05), and encouragement of autonomy (t = −5.09, β = −0.48, *p* < 0.05) negatively predicted social anxiety, which means that by increasing one unit in care, overprotecting, and encouragement of autonomy, the severity of social anxiety increased by 0.34, 0.19 and 0.34, respectively, but indifference was not a significant predictor of social anxiety (*p* > 0.05). 

Table 4 shows that components of paternal bonding were able to explain 27% of changes in social anxiety disorder (F = 21.52, *p* < 0.05). The results of the regression coefficients show that components such as care (t = −5.01, β = −0.35, *p* < 0.05), overprotecting (t = −2.77, β = −0.19, *p* < 0.05), and encouragement of autonomy (t = −5.09, β = −0.34, *p* < 0.05) negatively predicted social anxiety. This means that by increasing one unit in these variables, the numbers of social anxiety increased by 0.34, 0.19, and 0.34, respectively. But indifference was not a significant predictor of social anxiety (*p* > 0.05). 

As shown in Table 5, general components of fatherhood would explain 28% of changes in social anxiety (F = 10.16, *p* < 0.05). The factors like moral father role (t = −3.03, β = −0.21, *p* < 0.05), gender role model (t = −2.88, β = −0.25, *p* < 0.05), good provider role (t = −2.20, β = −0.15, *p* < 0.05), responsible father (t = −2.86, β = −0.22, *p* <.05), and accessible father (t = −4.20, β = −0.42, *p* < 0.05) negatively predicted social anxiety, which means that by increasing one unit in these subscales, the number of social anxiety decreased by 0.21, 0.25, 0.15, 0.22, and 0.41, respectively. Furthermore, based on regression coefficients, negative engagement (t = 4.38, β = 0.30, *p* < 0.05) positively predicted social anxiety, which means that by increasing one unit in negative engagement, the number of social anxiety increased by 0.30 units, but subscales like positive engagement, emotional responsiveness, and androgynous role were not significant predictors of social anxiety (*p* > 0.05). 

According to Table 6, components of adverse childhood experiences predicted 32% of changes in social anxiety (F = 22.01, *p* < 0.05). Based on the results of factors like experiences, components like physical abuse (t = 5.57, β = 0.32, *p* < 0.05), emotional abuse (t = 2.69, β = 0.18, *p* = 0.008), and sexual abuse (t = 4.43, β = 0.30, *p* < 0.05) positively predicted social anxiety. Therefore, by increasing one unit in these components, the number of social anxieties increased by 0.32, 0.18, and 0.28, respectively. But the components of physical and emotional neglect were not significant predictors of social anxiety (*p* > 0.05). 

## 4. Discussion

Social anxiety disorder is a deliberative disorder affecting a person’s social interactions throughout life [95]. It is crucial to investigate its etiology to prevent youth from developing symptoms as adults [52]. Families are the fundamental setting of a person’s existence; hence, their crucial effect on the etiology of social anxiety disorder must be studied [96]. The purpose of this study was to examine the predictive value of ACEs, parental bonding, and fatherhood concerning social anxiety symptoms. The study’s findings revealed that maternal and paternal bonding and fatherhood negatively predicted social anxiety, while ACEs positively predicted it. Also, among the components of ACEs, physical, emotional, and sexual abuse significantly and positively predicted social anxiety levels. These findings are in line with research by Meng et al. [31] and Myers and Liera [97]. Prior research centered on the effects of physical, sexual, and emotional abuse and viewed them as maltreatment, which can be an important risk factor for social anxiety [22]. Several plausible explanations exist for the observed relationships. Recent research indicates that physical, sexual, and particularly emotional abuse predict negative cognitions associated with anxiety disorders [98]. Moreover, among all forms of childhood maltreatment, emotional abuse significantly predicted difficulties with emotion regulation [41], which is highly related to social anxiety disorder [22].

Additionally, the current study found that maternal bonding, care, and encouragement of autonomy are associated with social anxiety. These findings are in line with the findings of Rambau et al. [99]. To illustrate, an intimate relationship with parents is an important factor that influences people’s socio-emotional function [100]. Studies have demonstrated the significance of a relationship with one’s parents in the development of behavioral problems [101]. A close relationship with parents can be a protective factor for children, helping to develop social skills and reducing social anxiety [102]. Also, high levels of mother–child closeness result in less social distress [103]. It appears that a close relationship with mothers improves a person’s cognitive and emotional regulation skills [104] and it enables adaptive coping with anxiety in a social context by creating a supportive environment and fostering social skills [105]. On the other hand, mothers’ behaviors like the encouragement of autonomy and overprotection might be related to some changes in the onset and maintenance of social anxiety [106]. These kinds of parental behaviors may become an impediment to normal development by preventing the child from engaging in age-appropriate activities, discouraging independence and autonomy, and encouraging dependency [107]. Such parents may unintentionally increase the risks of social anxiety by preventing their children from fully engaging in anxious situations, allowing them to develop an ineffective cognitive bias toward fearful stimuli [108].

The current study indicated that components of fatherhood, such as the moral father role, gender role model, good provider role, responsible father, accessible father, and negative engagement of the father, predicted social anxiety. The findings were in line with the results of Wang et al. [109] and Van Zalk et al. [83]. A meta-analysis by Yap and Jorm [110] supported the idea that fatherhood plays a vital role in predicting social anxiety. Studies in the field of developmental psychopathology revealed that if fathers had anxiety instead of intimacy, the child would be at risk of social anxiety. Also, negative paternal behavior such as criticism or rejection, developing a threatening view of the world, and preventing children from learning emotional management on their own is regarded as one of the most important causes of social anxiety [74]. Thus, it is no surprise that fathers’ negative behaviors can influence the onset and maintenance of social anxiety.

Additionally, high levels of father accessibility promote secure attachments, which reduce social anxiety [111]. Father’s accessibility may positively affect a person’s perception, e.g., when individuals know they can share their personal problems with their father or rely on his support in a difficult time, they believe they can act better in stressful situations due to his acceptance and protection; subsequently, this increases positive self-assessments in social interactions and decreases social anxiety levels [112].

## 5. Limitations and Future Directions

This study presents valuable results concerning the etiology of social anxiety. However, some limitations must be acknowledged. First, only emerging adults aged 18–29 living in a major metropolitan area (Tabriz) were included in the present study; thus, it is important to proceed with caution when applying these findings to different developmental stages or adults living in rural regions. Future studies should extend beyond emerging adults aged 18–29 in urban Tabriz to include diverse age groups and rural populations. This broader approach will provide a more comprehensive understanding of how parental bonding perceptions relate to social anxiety severity across different demographic segments within Iranian culture. Second, the risk of experiencing trauma during childhood exists for every individual, and these traumas can manifest themselves in multiple adverse experiences during childhood [113,114]. In other words, a person may simultaneously experience several traumatic events. Accordingly, participants may distort their memories either positively or negatively due to the passage of time or cognitive distortion, and the memories they have recalled during responses might not be exactly correct [115]. In the present study, participants between the ages of 18 and 29 had to recall adverse childhood experiences, parental bonding, and fatherhood during their middle childhood to early adolescence. Therefore, there is a possibility that the results may be affected by cognitive distortions rather than based on reality alone. Third, in this study, a retrospective self-report method was applied to assess the data rather than the multi-informant approach. The data were derived solely from participants’ self-reports. Self-report data are susceptible to response bias, and the responses were not confirmed by their family members or relatives [116]. Fourth, due to the cross-sectional nature of the study and the limited data from a homogenous population, causal relationships between social anxiety and other study variables cannot be precisely inferred. It is possible that other variables, such as social support and personality traits, can also contribute to the development of social anxiety; thus, future research should involve social support and personality qualities. Fifth, given that the majority of participants in this study were male, future studies with a majority of females could be conducted to determine whether the results would differ. Sixth, the findings of the present study may be specific to Iranian culture and may not generalize to other cultural contexts, limiting the external validity of the study. Overall, the study suggests practical implications such as developing targeted parental education programs, integrating findings into clinical interventions for social anxiety in Iranian contexts, advocating for supportive policies, raising community awareness about parenting impacts on mental health, training culturally sensitive mental health professionals, and conducting further longitudinal research to deepen understanding across developmental stages in Iranian society. These actions aim to enhance familial relationships and mitigate social anxiety severity through informed interventions and community support.

## 6. Conclusions

These findings suggest that adverse childhood experiences may significantly contribute to adult social anxiety. Additionally, fatherhood and parental bonding emerge as influential determinants of social anxiety in these individuals. The novelty of this work lies in its examination within the Iranian context, where cultural beliefs about fathers’ roles as dominant figures and the expectation that they should maintain distance from their children can strain father–child relationships, potentially exacerbating anxiety symptoms. Moreover, as fathers shift roles to assume responsibilities traditionally associated with mothers, such as caregiving and emotional support, new dynamics in parent–child relationships are emerging in Iranian society. This shift can impact familial interactions and, consequently, influence the development of social anxiety symptoms among individuals affected by adverse childhood experiences. It is important to note the current deficiency in interventions addressing these specific issues within the Iranian context, highlighting a significant gap in mental health support. Community-based programs, whether in societal or academic settings, play a crucial role in promoting mental health and fostering supportive environments. By targeting familial and societal influences identified in this study, such interventions aim to enhance resilience and mental well-being among individuals affected by ACEs and social anxiety.

## Figures and Tables

**Table 1:** Summary table of studies validating the psychometric properties of the AnTI, CTQ, PBI, and FS in different populations.

Tool	Author	Country	Participants	Reliability	Mean	SD ^a^	Reliability ^c^	Mean ^c^	SD ^c^
The Anxious Thought Inventory (AnTI)	[88]	England	*n*_1_ = 98 (41% male) *M (SD)_age_* = 21.22 (5.41) *n*_2_ = 141 (59% female) *M (SD)_age_* = 17.84 (2.37)	SW (*n*_1_, *n*_2_) = 0.84	SW (*n*_1_, *n*_2_) = 17.16, 19.12	SW (*n*_1_, *n*_2_) = 4.09, 4.95	SW (*n*) = 0.87	SW (*n*) = 16.98	SW (*n*) = 5.33
Childhood Trauma Questionnaire (CTQ)	[90]	United States	*n*_1_*=* 227 (57% female) *n*_2_ = 171 (43% male) *M (SD)_age_* = 14.9 (1.4)	EA (*n*_1_, *n*_2_) = 0.95 EN (*n*_1_, *n*_2_) = 0.94 SA (*n*_1_, *n*_2_) = 0.91 PA (*n*_1_, *n*_2_) = 0.90 PN (*n*_1_, *n*_2_) = 0.81 TS (*n*_1_, *n*_2_) = 0.97	EA (*n*_1_, *n*_2_) = 42.1 EN (*n*_1_, *n*_2_) = 52.8 SA (*n*_1_, *n*_2_) = 11.3 PA (*n*_1_, *n*_2_) = 13.4 PN (*n*_1_, *n*_2_) = 16.8 TS (*n*_1_, *n*_2_) = 136.5	EA (*n*_1_, *n*_2_) = 17.2 EN (*n*_1_, *n*_2_) = 19.5 SA (*n*_1_, *n*_2_) = 7.3 PA (*n*_1_, *n*_2_) = 7.1 PN (*n*_1_, *n*_2_) = 6.9 TS (*n*_1_, *n*_2_) = 47.0	EA (*n*) = 0.73 EN (*n*) = 0.83 SA (*n*) = 0.78 PA (*n*) = 0.81 PN (*n*) = 0.45 TS (*n*) = 0.57	EA (*n*) = 7.23 EN (*n)* = 9.68 SA (*n*) = 5.69 PA (*n*) = 5.72 PN (*n*) = 6.42 TS (*n*) = 34.76	EA (*n*) = 3.14 EN (*n*) = 4.32 SA (*n*) = 1.75 PA (*n*) = 1.87 PN (*n*) = 2.02 TS (*n*) = 9.81
Parental Bonding Instrument (PBI)	[91]	Australia	*n*_1_ = 79 (52.6% female) *n*_2_ = 71 (47.3% male) *M (SD)_age_* = 25	C (*n*_1_, *n*_2_) = 0.76 O (*n*_1_, *n*_2_) = 0.63 TPBS (*n*_1_, *n*_2_) = 0.70	C (*n*_1_, *n*_2_) = 24.9 O (*n*_1_, *n*_2_) = 13.3	b	C (*n*) = 0.79 O (*n*) = 0.73	C (*n*) = 20.87 O (*n*) = 20.61	C (*n*) = 3.72 O (*n*) = 4.52
Fatherhood Scale (FS)	[69]	United States	*n* = 311 (male) *M (SD)_age_* = 34 (10.5)	PER (*n*) = 0.96 PE (*n*) = 0.93 NE (*n*) = 0.85 MFR (*n*) = 0.86 GPR (*n*) = 0.90 GRM (*n*) = 0.80 AR (*n*) = 0.83 PA (*n*) = 0.87 PR (*n*) = 0.90 TFS (*n*) = 0.98	PER (*n*) = 57.0 PE (*n*) = 36.9 NE (*n*) = 48.2 MFR (*n*) = 14.6 GPR (*n*) = 19.4 GRM (*n*) = 16.1 AR (*n*) = 19.9 PA (*n*) = 10.2 PR (*n*) =27.8 TFS (*n*) = 232.9	PER (*n*) = 18.3 PE (*n*) = 13.6 NE (*n*) = 8.09 MFR (*n*) = 5.68 GPR (*n*) = 5.54 GRM (*n*) = 5.29 AR (*n*) = 5.51 PA (*n*) = 3.95 PR (*n*) = 10.0 TFS (*n*) = 54.3	PER (*n*) = 0.94 PE (*n*) = 0.89 NE (*n*) = 0.87 MFR (*n*) = 0.69 GPR (*n*) = 0.71 GRM (*n*) = 0.71 AR (*n*) = 0.72 PA (*n*) = 0.81 PR (*n*) = 0.73	PER (*n*) = 36.73 PE (*n*)= 12.92 NE (*n*) = 15.9 MFR (*n*) = 11.80 GPR (*n*) = 13.7 GRM (*n*) = 14.35 AR (*n*) = 16.39 PA (*n*) = 10.07 PR (*n*) = 17.56	PER (*n*) = 9.61 PE (*n*) = 3.9 NE (*n*) = 4.72 MFR (*n*) = 2.98 GPR (*n*) = 2.26 GRM (*n*) = 3.54 AR (*n*) = 3.54 PA (*n*) = 2.90 PR (*n*) = 4.34

Note. *n* = sample size. SW = social worry; EA = emotional abuse; EN = emotional neglect; SA = sexual abuse; PA = physical abuse; PN = physical neglect; TS = total scale; PER = positive emotion responsiveness; PE = positive engagement; NE = negative engagement; MFR = moral father role; GPR = good provider roll; GRM = gender role model; AR = androgynous role; PA = paternal accessibility; PR = paternal responsibility; TFS = total fatherhood scale; C = care; O = overprotection. a = Cronbach’s alpha and descriptive statistics of the original study. b = not reported in the original study. c = Cronbach’s alpha and descriptive statistics of the current study.

**Table 2:** Results of simultaneous multiple regressions for predicting social anxiety via total scores of maternal and paternal bonding, fatherhood, and adverse childhood experiences.

Variable	B	SE	β	T	Sig.	R	R^2^	F
Maternal model	−0.12	0.04	−0.20	−3.13	0.001	0.62	0.39	37.4
Fatherhood	−0.03	0.01	−0.12	−2.35	0.02			
Adverse childhood experience	0.13	0.04	0.22	3.48	0.001			

**Table 3:** Results of simultaneous multiple regression for predicting social anxiety via dimensions of maternal Bonding.

Variable	B	SE	β	T	Sig.	R	R^2^	F
Care	−0.28	0.12	−0.24	−2.33	0.02	0.51	0.26	20.70
Overprotection	−0.26	0.15	−0.18	−1.75	0.08			
Indifference	0.05	0.08	0.03	0.43	0.67			
Encouragement of autonomy	−0.52	0.08	−0.48	−6.41	0.001			

**Table 4:** Results of simultaneous multiple regression for predicting social anxiety via dimensions of paternal bonding.

Variable	B	SE	β	T	Sig.	R	R^2^	F
Care	−0.46	0.091	−0.35	−5.01	0.001	0.52	0.26	21.52
Overprotection	−0.24	0.08	−0.19	−2.77	0.006			
Indifference	−0.07	0.08	−0.06	−0.88	0.380			
Encouragement of autonomy	−0.43	0.08	−0.34	−5.09	0.001			

**Table 5:** Results of simultaneous multiple regression for predicting social anxiety via dimensions of fatherhood.

Variable	B	SE	β	T	Sig.	R	R^2^	F
Positive engagement	−0.13	0.124	−0.09	−1.07	0.28	0.53	0.28	10.16
Emotional responsiveness	0.01	0.045	0.02	0.25	0.80			
Negative engagement	0.25	0.057	0.30	4.38	0.001			
Moral father role	−0.30	0.097	−0.21	−3.03	0.001			
Gender role model	−0.31	0.11	−0.25	−2.88	0.001			
Good provider role	−0.29	0.13	−0.15	−2.20	0.03			
Androgynous role	0.005	0.11	0.004	0.05	0.96			
Responsible father	−0.28	0.097	−0.22	−2.86	0.005			
Accessible father	−0.76	0.18	0.42	−4.20	0.001			

**Table 6:** Results of simultaneous multiple regression for predicting social anxiety via dimensions of adverse childhood experiences.

Variable	B	SE	β	T	Sig.	R	R^2^	F
Physical abuse	0.80	0.14	0.32	5.57	0.001	0.564	0.32	22.01
Emotional abuse	0.33	0.12	0.18	2.70	0.008			
Sexual abuse	0.82	0.20	0.30	4.32	0.001			
Physical neglect	0.13	0.12	0.07	1.02	0.30			
Emotional neglect	0.07	0.07	0.06	0.90	0.37			

## Data Availability

The raw data supporting the conclusions of this article can be provided by the corresponding author upon reasonable request.

## References

[1] Stein M.B., Fuetsch M., Müller N., Höfler M., Lieb R., Wittchen H.-U. (2001). Social anxiety disorder and the risk of depression: A prospective community study of adolescents and young adults. Arch. Gen. Psychiatry.

[2] Burstein M., He J.-P., Kattan G., Albano A.M., Avenevoli S., Merikangas K.R. (2011). Social phobia and subtypes in the National Comorbidity Survey–Adolescent Supplement: Prevalence, correlates, and comorbidity. J. Am. Acad. Child Adolesc. Psychiatry.

[3] Wagner G., Zeiler M., Waldherr K., Philipp J., Truttmann S., Dür W., Treasure J.L., Karwautz A.F. (2017). Mental health problems in Austrian adolescents: A nationwide, two-stage epidemiological study applying DSM-5 criteria. Eur. Child Adolesc. Psychiatry.

[4] Knappe S., Beesdo-Baum K., Fehm L., Stein M.B., Lieb R., Wittchen H.-U. (2011). Social fear and social phobia types among community youth: Differential clinical features and vulnerability factors. J. Psychiatr. Res..

[5] Jefferies P., Ungar M. (2020). Social anxiety in young people: A prevalence study in seven countries. PLoS ONE.

[6] Talepasand S., Nokani M. (2010). Social phobia symptoms: Prevalence and sociodemographic correlates. Arch. Iran. Med..

[7] Mazhari S., Ekhlaspour M., Banazadeh N. (2014). Social Phobia and its Association with Academic Performance among Student of Kerman University of Medical Sciences Iran. Strides Dev. Med. Educ..

[8] Scharfstein L., Alfano C., Beidel D., Wong N. (2011). Children with generalized anxiety disorder do not have peer problems, just fewer friends. Child Psychiatry Hum. Dev..

[9] Lau N., Zhou A.M., Yuan A., Parigoris R., Rosenberg A.R., Weisz J.R. (2022). Social skills deficits and self-appraisal biases in children with social anxiety disorder. J. Child Fam. Stud..

[10] Amin R., Svedberg P., Narusyte J. (2019). Associations between adolescent social phobia, sickness absence and unemployment: A prospective study of twins in Sweden. Eur. J. Public Health.

[11] McNeil D.W. (2001). Terminology and Evolution of Constructs in Social Anxiety and Social Phobia.

[12] Lee H.Y., Kim I., Nam S., Jeong J. (2020). Adverse childhood experiences and the associations with depression and anxiety in adolescents. Child. Youth Serv. Rev..

[13] Crouch E., Probst J.C., Radcliff E., Bennett K.J., McKinney S.H. (2019). Prevalence of adverse childhood experiences (ACEs) among US children. Child Abus. Negl..

[14] Wang D., Jiang Q., Yang Z., Choi J.-K. (2021). The longitudinal influences of adverse childhood experiences and positive childhood experiences at family, school, and neighborhood on adolescent depression and anxiety. J. Affect. Disord..

[15] Felitti V.J., Anda R.F., Nordenberg D., Williamson D.F., Spitz A.M., Edwards V., Marks J.S. (1998). Relationship of childhood abuse and household dysfunction to many of the leading causes of death in adults: The Adverse Childhood Experiences (ACE) Study. Am. J. Prev. Med..

[16] Hargreaves M.K., Mouton C.P., Liu J., Zhou Y.E., Blot W.J. (2019). Adverse childhood experiences and health care utilization in a low-income population. J. Health Care Poor Underserved.

[17] Asmussen K., Fischer F., Drayton E., McBride T. (2020). Adverse childhood experiences: What we know, what we don’t know, and what should happen next. Early Interv. Found..

[18] Baheshmat S., Gholami J., Amin-Esmaeili M., Shadloo B., Rahimi-Movaghar A. (2022). Spouse and child abuse associated with illicit drug use in iran: A systematic review and meta-analysis. Trauma Violence Abus..

[19] Zarafshan H., Wissow L.S., Shahrivar Z., Mojtabai R., Khademi M., JafariNia M., Hajebi A., Abolhassani F., Sharifi V. (2021). Children and adolescents’ mental health in Iran’s primary care: Perspectives of general practitioners, school staff, and help seekers. Glob. Soc. Welf..

[20] Karbasi Z., Kadivar M., Safdari R., Shahmoradi L., Zahmatkeshan M., Zakerabasali S., Abhari S., Sayarifard A. (2020). Better monitoring of abused children by designing a child abuse surveillance system: Determining national child abuse minimum data set. Int. J. Health Plan. Manag..

[21] Kessler R.C., McLaughlin K.A., Green J.G., Gruber M.J., Sampson N.A., Zaslavsky A.M., Aguilar-Gaxiola S., Alhamzawi A.O., Alonso J., Angermeyer M. (2010). Childhood adversities and adult psychopathology in the WHO World Mental Health Surveys. Br. J. Psychiatry.

[22] Fitzgerald M., Gallus K. (2020). Emotional support as a mechanism linking childhood maltreatment and adult’s depressive and social anxiety symptoms. Child Abus. Negl..

[23] Crandall A., Broadbent E., Stanfill M., Magnusson B.M., Novilla M.L.B., Hanson C.L., Barnes M.D. (2020). The influence of adverse and advantageous childhood experiences during adolescence on young adult health. Child Abus. Negl..

[24] Chapman D.P., Dube S.R., Anda R.F. (2007). Adverse childhood events as risk factors for negative mental health outcomes. Psychiatr. Ann..

[25] Kim Y., Lee H., Park A. (2021). Patterns of adverse childhood experiences and depressive symptoms: Self-esteem as a mediating mechanism. Soc. Psychiatry Psychiatr. Epidemiol..

[26] Schalinski I., Teicher M.H., Nischk D., Hinderer E., Müller O., Rockstroh B. (2016). Type and timing of adverse childhood experiences differentially affect severity of PTSD, dissociative and depressive symptoms in adult inpatients. BMC Psychiatry.

[27] DeLisi M., Drury A.J., Elbert M.J. (2019). The etiology of antisocial personality disorder: The differential roles of adverse childhood experiences and childhood psychopathology. Compr. Psychiatry.

[28] Rogers C.J., Pakdaman S., Forster M., Sussman S., Grigsby T.J., Victoria J., Unger J.B. (2022). Effects of multiple adverse childhood experiences on substance use in young adults: A review of the literature. Drug Alcohol Depend..

[29] Longobardi C., Badenes-Ribera L., Fabris M.A. (2022). Adverse childhood experiences and body dysmorphic symptoms: A meta-analysis. Body Image.

[30] Li M., D’Arcy C., Meng X. (2016). Maltreatment in childhood substantially increases the risk of adult depression and anxiety in prospective cohort studies: Systematic review, meta-analysis, and proportional attributable fractions. Psychol. Med..

[31] Meng T., He Y., Zhang Q., Yu F., Zhao L., Zhang S., Chen Z., Wang S., Gong J., Liu J. (2021). Analysis of features of social anxiety and exploring the relationship between childhood major adverse experiences and social anxiety in early adulthood among Chinese college students. J. Affect. Disord..

[32] Watt T., Ceballos N., Kim S., Pan X., Sharma S. (2020). The unique nature of depression and anxiety among college students with adverse childhood experiences. J. Child Adolesc. Trauma.

[33] Bandoli G., Campbell-Sills L., Kessler R.C., Heeringa S.G., Nock M.K., Rosellini A.J., Sampson N.A., Schoenbaum M., Ursano R.J., Stein M.B. (2017). Childhood adversity, adult stress, and the risk of major depression or generalized anxiety disorder in US soldiers: A test of the stress sensitization hypothesis. Psychol. Med..

[34] Cisler J.M., Olatunji B.O., Feldner M.T., Forsyth J.P. (2010). Emotion regulation and the anxiety disorders: An integrative review. J. Psychopathol. Behav. Assess..

[35] Domaradzka E., Fajkowska M. (2018). Cognitive emotion regulation strategies in anxiety and depression understood as types of personality. Front. Psychol..

[36] Gross J.J. (2002). Emotion regulation: Affective, cognitive, and social consequences. Psychophysiology.

[37] Gratz K.L., Roemer L. (2004). Multidimensional assessment of emotion regulation and dysregulation: Development, factor structure, and initial validation of the difficulties in emotion regulation scale. J. Psychopathol. Behav. Assess..

[38] Webster E.M. (2022). The impact of adverse childhood experiences on health and development in young children. Glob. Pediatr. Health.

[39] Miu A.C., Szentágotai-Tătar A., Balazsi R., Nechita D., Bunea I., Pollak S.D. (2022). Emotion regulation as mediator between childhood adversity and psychopathology: A meta-analysis. Clin. Psychol. Rev..

[40] Buhle J.T., Silvers J.A., Wager T.D., Lopez R., Onyemekwu C., Kober H., Weber J., Ochsner K.N. (2014). Cognitive reappraisal of emotion: A meta-analysis of human neuroimaging studies. Cereb. Cortex.

[41] Burns E.E., Jackson J.L., Harding H.G. (2010). Child maltreatment, emotion regulation, and posttraumatic stress: The impact of emotional abuse. J. Aggress. Maltreatment Trauma.

[42] Heimberg R.G., Brozovich F.A., Rapee R.M. (2014). A cognitive-behavioral model of social anxiety disorder. Soc. Anxiety.

[43] Heimberg R.G., Brozovich F.A., Rapee R.M., Hofmann S.G., DiBartolo P.M. (2010). Chapter 15—A Cognitive Behavioral Model of Social Anxiety Disorder: Update and Extension. Social Anxiety.

[44] Bowlby J. (1973). Attachment and Loss. Volume II. Separation, Anxiety and Anger.

[45] Stiller J., Alfermann D., Liukkonen J., Vanden Auweele Y., Vereijken B., Alfermann D., Theodorakis Y. (2007). Promotion of a Healthy Self-Concept. Psychology for Physical Educators: Student in Focus.

[46] Liu R.T., Kleiman E.M., Nestor B.A., Cheek S.M. (2015). The hopelessness theory of depression: A quarter-century in review. Clin. Psychol. Sci. Pract..

[47] Westermair A.L., Stoll A.M., Greggersen W., Kahl K.G., Hüppe M., Schweiger U. (2018). All unhappy childhoods are unhappy in their own way—Differential impact of dimensions of adverse childhood experiences on adult mental health and health behavior. Front. Psychiatry.

[48] Vaughn M.G., Salas-Wright C.P., Huang J., Qian Z., Terzis L.D., Helton J.J. (2017). Adverse childhood experiences among immigrants to the United States. J. Interpers. Violence.

[49] Essau C.A., Conradt J., Sasagawa S., Ollendick T.H. (2012). Prevention of anxiety symptoms in children: Results from a universal school-based trial. Behav. Ther..

[50] Ho G.W., Bressington D., Karatzias T., Chien W.-T., Inoue S., Yang P.J., Chan A.C., Hyland P. (2020). Patterns of exposure to adverse childhood experiences and their associations with mental health: A survey of 1346 university students in East Asia. Soc. Psychiatry Psychiatr. Epidemiol..

[51] Kim Y., Park A., Murphy J. (2023). Patterns of adverse childhood experiences and mental health: Evidence from college students in Korea. J. Interpers. Violence.

[52] Spence S.H., Rapee R.M. (2016). The etiology of social anxiety disorder: An evidence-based model. Behav. Res. Ther..

[53] Tichelman E., Westerneng M., Witteveen A.B., Van Baar A.L., Van Der Horst H.E., De Jonge A., Berger M.Y., Schellevis F.G., Burger H., Peters L.L. (2019). Correlates of prenatal and postnatal mother-to-infant bonding quality: A systematic review. PLoS ONE.

[54] Liu Y.-L. (2008). An examination of three models of the relationships between parental attachments and adolescents’ social functioning and depressive symptoms. J. Youth Adolesc..

[55] Wu C.-h. (2009). The relationship between attachment style and self-concept clarity: The mediation effect of self-esteem. Personal. Individ. Differ..

[56] Engels R.C., Finkenauer C., Meeus W., Deković M. (2001). Parental attachment and adolescents’ emotional adjustment: The associations with social skills and relational competence. J. Couns. Psychol..

[57] Field N.P., Om C., Kim T., Vorn S. (2011). Parental styles in second generation effects of genocide stemming from the Khmer Rouge regime in Cambodia. Attach. Hum. Dev..

[58] Nanda M.M., Reichert E., Jones U.J., Flannery-Schroeder E. (2016). Childhood maltreatment and symptoms of social anxiety: Exploring the role of emotional abuse, neglect, and cumulative trauma. J. Child Adolesc. Trauma.

[59] Breidenstine A.S., Bailey L.O., Zeanah C.H., Larrieu J.A. (2011). Attachment and trauma in early childhood: A review. J. Child Adolesc. Trauma.

[60] Mak H.W., Fosco G.M., Feinberg M.E. (2018). The role of family for youth friendships: Examining a social anxiety mechanism. J. Youth Adolesc..

[61] Frosch C.A., Schoppe-Sullivan S.J., O’Banion D.D. (2021). Parenting and Child Development: A Relational Health Perspective. Am. J. Lifestyle Med..

[62] Morris T.L., Oosterhoff B. (2016). Observed mother and father rejection and control: Association with child social anxiety, general anxiety, and depression. J. Child Fam. Stud..

[63] Kidd K.N., Prasad D., Cunningham J.E., de Azevedo Cardoso T., Frey B.N. (2022). The relationship between parental bonding and mood, anxiety and related disorders in adulthood: A systematic review and meta-analysis. J. Affect. Disord..

[64] Dong Z., Zhou S., Case A.S., Zhou W. (2022). The Relationship Between Perceived Parenting Style and Social Anxiety: A Meta-analysis of Mainland Chinese Students. Child Psychiatry Hum. Dev..

[65] Dixon M.L., Moodie C.A., Goldin P.R., Farb N., Heimberg R.G., Gross J.J. (2020). Emotion regulation in social anxiety disorder: Reappraisal and acceptance of negative self-beliefs. Biol. Psychiatry Cogn. Neurosci. Neuroimaging.

[66] Arditti J.A. (2012). Child trauma within the context of parental incarceration: A family process perspective. J. Fam. Theory Rev..

[67] Burns A., Leavey G., O’Sullivan R. (2022). Associations between parental bonding, social isolation and loneliness: Do associations persist in later life and is isolation a mediator between parental bonding and loneliness?. BMC Psychol..

[68] Bögels S.M., Bamelis L., van der Bruggen C. (2008). Parental rearing as a function of parent’s own, partner’s, and child’s anxiety status: Fathers make the difference. Cogn. Emot..

[69] Dick G.L. (2004). The fatherhood scale. Res. Soc. Work Pract..

[70] Lester L., Pearce N., Waters S., Barnes A., Beatty S., Cross D. (2017). Family involvement in a whole-school bullying intervention: Mothers’ and fathers’ communication and influence with children. J. Child Fam. Stud..

[71] Chorpita B.F., Albano A.M., Barlow D.H. (1996). Cognitive processing in children: Relation to anxiety and family influences. J. Clin. Child Psychol..

[72] Lindhout I., Markus M., Hoogendijk T., Borst S., Maingay R., Spinhoven P., van Dyck R., Boer F. (2006). Childrearing style of anxiety-disordered parents. Child Psychiatry Hum. Dev..

[73] Kullberg M.-L., Maciejewski D., van Schie C.C., Penninx B.W., Elzinga B.M. (2020). Parental bonding: Psychometric properties and association with lifetime depression and anxiety disorders. Psychol. Assess..

[74] Bögels S., Phares V. (2008). Fathers’ role in the etiology, prevention and treatment of child anxiety: A review and new model. Clin. Psychol. Rev..

[75] Gutierrez-Galve L., Stein A., Hanington L., Heron J., Lewis G., O’Farrelly C., Ramchandani P.G. (2019). Association of maternal and paternal depression in the postnatal period with offspring depression at age 18 years. JAMA Psychiatry.

[76] Bandura A., Walters R.H. (1977). Social Learning Theory.

[77] Leidy M.S., Schofield T.J., Parke R.D. (2013). Fathers’ contributions to children’s social development. Handbook of Father Involvement.

[78] Paquette D. (2004). Theorizing the father-child relationship: Mechanisms and developmental outcomes. Hum. Dev..

[79] Bögels S.M., Perotti E.C. (2011). Does father know best? A formal model of the paternal influence on childhood social anxiety. J. Child Fam. Stud..

[80] Pougnet E., Serbin L.A., Stack D.M., Schwartzman A.E. (2011). Fathers’ influence on children’s cognitive and behavioural functioning: A longitudinal study of Canadian families. Can. J. Behav. Sci. Rev. Can. Des Sci. Du Comport..

[81] Gómez-Ortiz O., Romera E.M., Jiménez-Castillejo R., Ortega-Ruiz R., García-López L.J. (2019). Parenting practices and adolescent social anxiety: A direct or indirect relationship?. Int. J. Clin. Health Psychol..

[82] Suh G.W., Fabricius W.V., Stevenson M.M., Parke R.D., Cookston J.T., Braver S.L., Saenz D.S. (2016). Effects of the interparental relationship on adolescents’ emotional security and adjustment: The important role of fathers. Dev. Psychol..

[83] Van Zalk N., Tillfors M., Trost K. (2018). Mothers’ and fathers’ worry and over-control: One step closer to understanding early adolescent social anxiety. Child Psychiatry Hum. Dev..

[84] Rahkar Farshi M., Valizadeh L., Zamanzadeh V., Rssouli M., Lopez V., Cleary M. (2018). Perceptions of Iranian parents towards the paternal role in raising adolescent children. Nurs. Health Sci..

[85] Salami A., Ghajarieh A. (2016). Culture and gender representation in Iranian school textbooks. Sex. Cult..

[86] Tashakkori A., Mehryar A.H. (1982). The differential roles of parents in the family, as reported by a group of Iranian adolescents. J. Marriage Fam..

[87] Valizadeh S., Mirlashari J., Navab E., Higman W., Ghorbani F., Dowling D., Thibeau S. (2018). Fathers: The lost ring in the chain of family-centered care. Adv. Neonatal Care.

[88] Wells A. (1994). A multi-dimensional measure of worry: Development and preliminary validation of the Anxious Thoughts Inventory. Anxiety Stress Coping.

[89] Moloodi R., Dobson K., Fata L., Pourshahbaz A., Mohammadkhani P., Mootabi F., Kami M., Ziai K., Ghaderi A. (2020). Psychometric properties of Persian version of Cognitive Behavioural Avoidance Scale: Results from student, general population and clinical samples in Iran. Behav. Cogn. Psychother..

[90] Bernstein D.P., Ahluvalia T., Pogge D., Handelsman L. (1997). Validity of the Childhood Trauma Questionnaire in an adolescent psychiatric population. J. Am. Acad. Child Adolesc. Psychiatry.

[91] Parker G. (1990). The Parental Bonding Instrument: A decade of research. Soc. Psychiatry Psychiatr. Epidemiol. Int. J. Res. Soc. Genet. Epidemiol. Ment. Health Serv..

[92] Wilhelm K., Niven H., Parker G., Hadzi-Pavlovic D. (2005). The stability of the Parental Bonding Instrument over a 20-year period. Psychol. Med..

[93] Behzadi B., Parker G. (2015). A Persian version of the parental bonding instrument: Factor structure and psychometric properties. Psychiatry Res..

[94] Amlashi Z.E., Hosseinkhanzadeh A.A., Akbari B., Moghtader L. (2021). Psychometric properties of the parenting self-efficacy questionnaire (TOPSE). J. Psychol. Sci..

[95] Heerey E.A., Kring A.M. (2007). Interpersonal consequences of social anxiety. J. Abnorm. Psychol..

[96] Merikangas K., Lieb R., Wittchen H.U., Avenevoli S. (2003). Family and high-risk studies of social anxiety disorder. Acta Psychiatr. Scand..

[97] Myers N.S., Llera S.J. (2020). The role of childhood maltreatment in the relationship between social anxiety and dissociation: A novel link. J. Trauma Dissociation.

[98] Teicher M.H., Samson J.A., Anderson C.M., Ohashi K. (2016). The effects of childhood maltreatment on brain structure, function and connectivity. Nat. Rev. Neurosci..

[99] Rambau S., Forstner A.J., Wegener I., Mücke M., Wissussek C.T., Staufenbiel S.M., Geiser F., Schumacher J., Conrad R. (2018). Childhood adversities, bonding, and personality in social anxiety disorder with alcohol use disorder. Psychiatry Res..

[100] Thomas R., Abell B., Webb H.J., Avdagic E., Zimmer-Gembeck M.J. (2017). Parent-child interaction therapy: A meta-analysis. Pediatrics.

[101] Nishida A., Richards M., Stafford M. (2016). Prospective associations between adolescent mental health problems and positive mental wellbeing in early old age. Child Adolesc. Psychiatry Ment. Health.

[102] Early D.M., Iruka I.U., Ritchie S., Barbarin O.A., Winn D.-M.C., Crawford G.M., Frome P.M., Clifford R.M., Burchinal M., Howes C. (2010). How do pre-kindergarteners spend their time? Gender, ethnicity, and income as predictors of experiences in pre-kindergarten classrooms. Early Child. Res. Q..

[103] Metin Aslan Ö., Altinisik Y. (2022). The role of mother–child relationship between young children’s anxiety and social play. Early Child Dev. Care.

[104] Bernier A., Carlson S.M., Whipple N. (2010). From external regulation to self-regulation: Early parenting precursors of young children’s executive functioning. Child Dev..

[105] Acar I.H., Pérez-González S., Kutaka T.S., Yıldız S. (2019). Difficult temperament and children’s peer relations: The moderating role of quality of parent–child relationships. Early Child Dev. Care.

[106] Spokas M., Heimberg R.G. (2009). Overprotective parenting, social anxiety, and external locus of control: Cross-sectional and longitudinal relationships. Cogn. Ther. Res..

[107] McLeod B.D., Weisz J.R., Wood J.J. (2007). Examining the association between parenting and childhood depression: A meta-analysis. Clin. Psychol. Rev..

[108] Cooper-Vince C.E., Pincus D.B., Comer J.S. (2014). Maternal intrusiveness, family financial means, and anxiety across childhood in a large multiphase sample of community youth. J. Abnorm. Child Psychol..

[109] Wang M., Wu X., Wang J. (2021). Paternal and maternal harsh parenting and Chinese adolescents’ social anxiety: The different mediating roles of attachment insecurity with fathers and mothers. J. Interpers. Violence.

[110] Yap M.B.H., Jorm A.F. (2015). Parental factors associated with childhood anxiety, depression, and internalizing problems: A systematic review and meta-analysis. J. Affect. Disord..

[111] Cavanaugh A.M., Buehler C. (2016). Adolescent loneliness and social anxiety: The role of multiple sources of support. J. Soc. Pers. Relatsh..

[112] Sarkadi A., Kristiansson R., Oberklaid F., Bremberg S. (2008). Fathers’ involvement and children’s developmental outcomes: A systematic review of longitudinal studies. Acta Paediatr..

[113] McCann J.B., James A., Wilson S., Dunn G. (1996). Prevalence of psychiatric disorders in young people in the care system. BMJ.

[114] Nelson C.A., Scott R.D., Bhutta Z.A., Harris N.B., Danese A., Samara M. (2020). Adversity in childhood is linked to mental and physical health throughout life. BMJ.

[115] Schacter D.L., Kagan J., Leichtman M.D. (1995). True and false memories in children and adults: A cognitive neuroscience perspective. Psychol. Public Policy Law.

[116] McDonald J.D. (2008). Measuring personality constructs: The advantages and disadvantages of self-reports, informant reports and behavioural assessments. Enquire.

